# Autophagy Differentially Influences Toll-like Receptor 9 and B Cell-Receptor-Mediated B Cell Expansion, Expression of Major Histocompatibility Class II Proteins, and Antigenic Peptide Presentation

**DOI:** 10.3390/ijms26136054

**Published:** 2025-06-24

**Authors:** Chander Peddaboina, Jaclyn Iannucci, Richard P. Tobin, Lee A. Shapiro, M. Karen Newell Rogers

**Affiliations:** 1 Department of Medical Physiology, Texas A&M University College of Medicine, Bryan, TX 77807, USA; 2 Department of Neuroscience and Experimental Therapeutics, Texas A&M University College of Medicine, Bryan, TX 77807, USA; jmiannucci@tamu.edu; 3 Department of Surgery, Division of Surgical Oncology, University of Colorado Anschutz Medical Campus, Aurora, CO 80045, USA

**Keywords:** adaptive immunity, autophagy, innate immunity, macro-autophagy, Toll-like receptors

## Abstract

B cells contribute to innate and adaptive immunity. In the former, Toll-like receptor (TLR) activation promotes the expansion of inflammatory B cells. In the latter, B cell receptor (BCR) activation results in the production of antibodies or autoantibodies. Antigen processing and presentation are closely associated with major histocompatibility class II (MHC-II) and its companion protein, class II invariant peptide (CLIP). The impact of autophagy on the regulation of these unique mechanisms of B cell activation and subset expansion has not been fully explored. The results from the current study show that activating autophagy with rapamycin (RAPA) or inhibiting autophagy with hydroxycholoroquine (HCQ) differentially influences the TLR9 and BCR activation of B cells. These differences include the selective expansion of B1 and B2 B cell subsets, the regulation of the cell-surface expression of MHC-II and CLIP, and the ability of distinct B cell subsets to present peptide antigens. These novel findings demonstrate that the unique B cell activation mechanisms induced by TLR9 and BCR activation are differentially influenced by RAPA and HCQ, owing to the selective modulation of B cell subset expansion, and antigen processing and presentation by MHC-II proteins.

## 1. Introduction

B cells are critically involved in a variety of inflammatory, immune, and autoimmune diseases. Therapeutic targeting and depletion of B cells are beneficial in several disorders, including multiple sclerosis, type I diabetes, and rheumatoid arthritis [1]. The activation and selection of B cells are mediated by multiple mechanisms that are regulated by autophagy [2,3]. Bidirectionally, B cell activation can induce canonical or non-canonical autophagy to shape the B cell repertoire [4]. Autophagy is required for self-renewal of B1 B cells because the deletion of the Atg7 gene results in the failure of self-renewal of these cells as a result of mitochondrial and metabolic dysfunction [5]. While the B1 B cell subset is predominantly found in the peritoneum and other cavities and tends to produce non-specific innate-associated cytokines and chemokines, autophagy also regulates the development of conventional bone marrow-derived B2 B cells. These latter cells are crucial for increasing the repertoire of antibody-secreting cells and shaping the adaptive immune response [3,6,7,8]. Invariant chain (CD74), and its cleavage product, major histocompatibility complex (MHC) class II invariant peptide (CLIP), also play major roles in regulating mature B cell selection and survival [9,10,11]. While previous studies have shown that autophagy promotes the loading of intracellular peptides into the antigen-binding groove of MHC-II [12], it is still unknown whether autophagy can be modulated to influence CLIP in the groove of MHC-II.

Several mechanisms induce B cell activation, including stimulation of Toll-like receptors (TLR) and antigen binding to the B cell receptor (BCR). Autophagy and TLR or BCR B cell activation are bidirectionally linked. TLR activation induces autophagy that drives plasmoblast differentiation [4,13], whereas BCR-induced activation of autophagy may not be required to stimulate proliferation and differentiation [8,14]. Autophagy in turn also mediates activation by TLRs and BCR, and some of these mechanisms appear to be distinct and differentially regulated [4,15,16,17]. Among the TLRs that are expressed on a variety of cell types, including B lymphocytes [18], TLR9 is the receptor for cytidine monophosphate guanosine oligodeoxynucleotide (CpG) motifs [19]. TLR9 has been shown to initiate autophagy and autophagosome assembly [20,21,22,23], which mediate TLR9-induced innate immune [24,25] and adaptive immune autoantibody production [26,27]. While TLR activation has also been shown to increase cell-surface CLIP [28], it is unknown if modulating autophagy will impact TLR9-induced CLIP expression.

Previous studies have shown that autophagy influences B cell selection following BCR activation [5,29,30]. BCR activation has also been shown to increase CD74 [31], but its effects on CLIP and MHC-II expression have not been fully elucidated. The collective contributions of MHC-II and CLIP are key components of B cell function and play a major role in antigen processing and presentation [15,32]. Thus, the current study was designed to test the hypothesis that autophagy induction by rapamycin (RAPA) and autophagy inhibition by hydroxychloroquine (HCQ) will differentially regulate TLR9- or BCR-induced B cell subset expansion, CLIP, and MHC-II expression, and antigen presentation.

## 2. Results and Discussion

### 2.1. TLR9 and BCR Activation Drive Opposing B Cell Subsets

**Figure 1:**
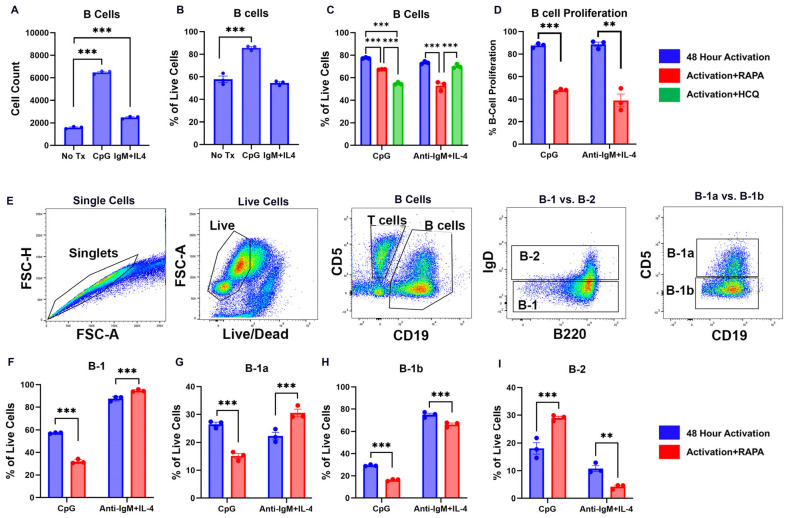
B cell proliferation in response to TLR9 and BCR stimulation. Splenocytes from C57bl6/J mice were incubated for 48 h in vitro, with either CpG to activate TLR9 or anti-IgM+IL-4 to activate BCR. There was a significant increase in the number of B cells with TLR9 and BCR activation (**A**). TLR9, but not BCR stimulation, significantly increased the percentage of B cells (**B**). In the TLR9-stimulated splenocytes, treatment with rapamycin (RAPA) or hydroxychloroquine (HCQ) significantly decreased the percentage of B cells (**C**). In the BCR-activated splenocytes, only RAPA significantly decreased the percentage of B Cells, while HCQ had no effect (**C**). RAPA also significantly decreased the proliferation of B cells in TLR9 and BCR-activated B cells (**D**). (**E**) Gating strategy for B-1, B-1a, B-1b, and B-2 cells. Following TLR9 activation, RAPA significantly decreased B-1 (**F**), B-1a (**G**), and B-1b (**H**) B cells and significantly increased B-2 B cells (**I**). Following BCR activation, RAPA resulted in a significant increase in the percentage of B-1 and B-1a B cells (**F**,**G**), whereas B-1b and B-2 cells were significantly decreased (**H**,**I**). Therefore, the influence of RAPA on TLR9-activated B cell subsets is to decrease the B1 subsets and increase B2, whereas RAPA generally had the opposite effect following BCR activation, increasing B1 and B1a, but decreasing B1b and B2 subsets. Data are presented as mean ± SEM, n = 3. ** *p* < 0.01, *** *p* < 0.001.

Splenocytes were treated for 48 h with either CpG, a TLR9 agonist, or the F(ab)’2 fragment of anti-mouse IgM in combination with IL-4 (anti-IgM+IL-4), as a surrogate for antigen-mediated BCR activation. To exclude Fc receptor engagement that can result in a negative signal via the Fc receptor, a fragment of the antibody against the B cell antigen receptor was used in lieu of the intact antibody [33]. B cell proliferation in response to either BCR or TLR9 activation was confirmed. TLR9 activation with CpG significantly increased the frequency of B cells (*p* < 0.001) and the total number of B cells (*p* < 0.001) (Figure 1A,B). By contrast, BCR engagement with anti-IgM+IL-4 significantly increased only the total number of B cells (*p* < 0.001) but not the frequency of B cells (Figure 1A,B).

The next experiments were designed to determine the role of autophagy induction by RAPA and the inhibition of autophagy by HCQ on B cell proliferation in response to TLR9 and BCR activation in B cells. RAPA or HCQ were introduced in vitro at the time of TLR or BCR activation. Consistent with previous studies, RAPA significantly reduced the percentage of total B cells in both CpG-treated (*p* < 0.001) and anti-IgM+IL-4-treated (*p* < 0.001) cells (Figure 1C) [34,35,36]. RAPA also significantly reduced B cell proliferation after 24 h in both CpG-treated (*p* < 0.001) and anti-IgM+IL-4-treated splenocytes (*p* < 0.01) (Figure 1D). HCQ significantly reduced the percentage of live B cells in CpG-activated splenocytes (*p* < 0.001) but not in anti-IgM+IL-4-treated cultures (Figure 1C), suggesting that HCQ impairs the TLR9-induced B cell expansion but not the BCR-induced expansion. Thus, there may be distinct autophagic mechanisms that regulate TLR9- and BCR-mediated B cell activation.

Because autophagy is involved in the self-renewal of B1 B cells [5], the next set of experiments examined the B cell repertoire after TLR9 and BCR activation and in response to autophagy induction with RAPA. Peritoneal B cells, which consist of a higher ratio of B1 to B2 B cells [37,38], were isolated and activated in vitro with CpG or anti-IgM+IL-4, with or without RAPA. Consistent with previous findings [39,40], RAPA significantly reduced the frequency of IgD^−^B220^+^/CD19^+^ B-1 B cells (Figure 1F; *p* < 0.001) and significantly increased the frequency of IgD^+^B220^−^/CD19^+^ B-2 B cells (Figure 1I; *p* < 0.001). Further examination of the B-1 subsets showed that RAPA significantly reduced the frequency of both CD5^+^IgD^−^B220^+^/CD19^+^ B-1a (*p* < 0.001) and CD5^−^IgD^−^B220^+^/CD19^+^ B-1b (*p* < 0.001) cells after TLR9 activation (Figure 1G,H). In contrast, RAPA significantly increased IgD^−^B220^+^/CD19^+^ B-1 B cells subsets (Figure 1F; *p* < 0.001) and significantly decreased the frequency of IgD^+^B220^−^/CD19^+^ B-2 B cells (Figure 1I; *p* < 0.01) after BCR activation. RAPA also significantly increased the frequency of CD5^+^IgD^−^B220^+^/CD19^+^ B-1a cells (*p* < 0.001) but significantly reduced the frequency of CD5^−^IgD^−^B220^+^/CD19^+^ B-1b cells following BCR activation (*p* < 0.001; Figure 1G,H). Therefore, a major novel finding of this study is that TLR9 and BCR activation results in differential B cell selection, and autophagy induction with RAPA selectively decreases B-1a and B-1b cells following TLR9 activation but increases B-1a cells after BCR activation. These findings extend previous studies that have demonstrated mechanisms through which autophagy shapes the B cell repertoire following B cell activation.

### 2.2. TLR9 and BCR Activation Induce MHC-II and CLIP Expression on B Cells That Are Modified by RAPA and HCQ

**Figure 2:**
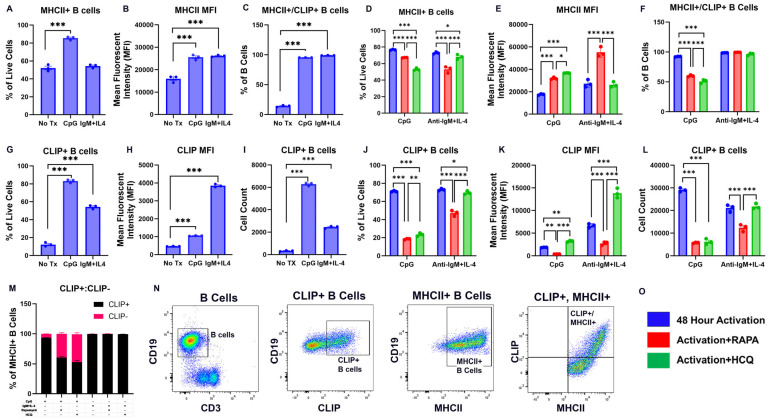
The effects of RAPA and HCQ on MHC-II and CLIP following B cell activation. TLR9 and BCR were activated in splenocytes, as shown in Figure 1. The percentage of MHC-II^+^ B cells was significantly increased only by TLR9 activation (**A**), although both TLR9 and BCR activation increased the MFI of MHC-II (**B**). Following TLR9 and BCR activation, RAPA and HCQ significantly decreased the percentage of MHC-II^+^ cells (**D**). RAPA and HCQ significantly increased the mean fluorescent intensity (MFI) of MHC-II following TLR9 activation (**E**), and RAPA significantly increased the MFI of MHC-II (**E**). As shown in (**G**–**I**), both CPG and anti-IgM+IL-4 increased the percentage of CLIP^+^ B cells, the MFI of CLIP, and the total number of CLIP^+^ B cells, respectively. The percentage of CLIP^+^ B cells was significantly decreased by RAPA and HCQ (**J**). The number and MFI of CLIP^+^ B cells were decreased by RAPA in both TLR9- and BCR-activated B cells (**K**,**L**), whereas HCQ significantly increased the MFI of CLIP (**K**). The ratio of MHC-II^+^/CLIP^+^ cells highlights the observation that both TLR9 and BCR activation increase MHC-II^+^/CLIP^+^ B cells (**C**), and RAPA significantly decreases these cells (**F**). Considering that RAPA decreased CLIP without influencing MHC-II (as shown by the increased CLIP-/MHC-II+ B cells in pink (**M**)) following TLR9 activation, RAPA has a potential role in the displacement of CLIP from the binding groove of MHC-II. In (**N**), the gating strategy used to identify B cells, CLIP^+^ B cells, and MHC-II^+^ B cells is shown. (**O**) Key for graphs in (**D**–**F**,**J**–**L**). Data are presented as mean ± SEM, n = 3. * *p* < 0.05, ** *p* < 0.01, *** *p* < 0.001.

CD74 is involved in the assembly of MHC-II molecules, and CD74 also serves as a chaperone for MHC-II migration to the cell surface [41]. CD74 can be proteolytically cleaved in the lysosome, resulting in the generation of the CLIP peptide. CLIP occupies the antigen-binding groove of cell-surface MHC-II, preventing peptide binding until displacement is facilitated by human leukocyte antigen DM (HLA-DM; mouse analog = H-2M) [42]. Considering that RAPA and HCQ had differential effects on B cell expansion, the next series of experiments were designed to determine how RAPA and HCQ regulate MHC-II on activated B cells. The percentage of MHC-II^+^ B cells was significantly increased by CpG (*p* < 0.001) (Figure 2A) but not in response to anti-IgM+IL-4. Both CpG and anti-IgM+IL-4 significantly increased MHC-II mean fluorescent intensity (MFI) on B cells (*p* < 0.001, *p* < 0.001, respectively) (Figure 2B). RAPA significantly reduced the percentage of MHC-II^+^ B cells in both CpG-treated (*p* < 0.001) and anti-IgM+IL-4-treated (*p* < 0.001) cells and significantly increased the MFI of MHC-II per cell, in both CpG-activated (*p* < 0.001) and anti-IgM+IL-4-activated (*p* < 0.001) cells (Figure 2D,E). That RAPA reduced the frequency of MHC-II^+^ B cells but increased the expression level of MHC-II on the remaining B cells could indicate that autophagy regulates the function of antigen or autoantigen processing and presentation in B cells. HCQ significantly decreased the percentage of MHC-II^+^ cells (*p* < 0.001) and significantly increased MHC-II MFI (*p* < 0.001) in CpG-stimulated cells (Figure 2D,E). Interestingly, HCQ did not affect MHC-II expression on B cells in anti-IgM+IL-4-stimulated cells (Figure 2E).

In its role in inhibiting autoimmunity, CLIP has been shown to prevent the binding of self-antigen [43,44]. The replacement of CLIP by antigenic peptide and subsequent presentation to CD4^+^ T cells results in the switch to an antigen-specific T cell-mediated adaptive immune response. Given the effects of RAPA and HCQ on MHC-II expression following TLR9 and BCR B cell activation, and the close relationship between MHC-II and CLIP, the next experiments evaluated the expression of CLIP in the groove of cell-surface MHC-II. There were significant increases in the percentage of CLIP^+^ B cells, the cell-surface expression of CLIP, as measured by MFI, and the total number of CLIP^+^ B cells in response to activation with either CpG (*p* < 0.001, *p* < 0.001, *p* < 0.001, respectively) or anti-IgM+IL-4 (*p* < 0.001, *p* < 0.001, *p* < 0.001, respectively; Figure 2G–I). This novel finding indicates that B cell activation by TLR9 or BCR engagement increases CLIP in the groove of MHC-II that is expressed on the cell surface.

Next, the role of autophagy induction by RAPA in the activation of CLIP^+^ B cells was investigated. RAPA significantly reduced the percentage of CLIP^+^ B cells after CpG (*p* < 0.001) and anti-IgM+IL-4 (*p* < 0.001) (Figure 2J). RAPA also significantly reduced the MFI of CLIP on B cells in both the CpG- and anti-IgM+IL-4-treated groups (Figure 2K; *p* < 0.01, *p* < 0.001, respectively), and the total number of CLIP^+^ B cells (Figure 2L; *p* < 0.001, *p* < 0.001). Therefore, RAPA reduced the number of CLIP^+^ B cells and reduced the level of CLIP expression on the B cell surface. Interestingly, HCQ also significantly reduced the frequency of CLIP^+^ B cells in CpG-activated (*p* < 0.001) and anti-IgM+IL-4-activated (*p* < 0.05) cells. However, this reduction with HCQ was significantly less than that found with RAPA in both CpG-treated (*p* < 0.01 vs. RAPA) and anti-IgM+IL-4-treated (*p* < 0.001 vs. RAPA) cells (Figure 2J). In contrast to RAPA, which decreased CLIP MFI, inhibiting autophagy with HCQ resulted in a significant increase in the MFI of cell-surface CLIP on B cells, in both CpG-activated (*p* < 0.01) and anti-IgM+IL-4-activated (*p* < 0.001) splenocytes (Figure 2K). Therefore, autophagy plays a role in regulating cell-surface CLIP expression, such that the expansion of CLIP^+^ B cells is reduced by inducing autophagy with RAPA, whereas CLIP expression on B cells is enhanced by inhibiting autophagy with HCQ.

### 2.3. RAPA and HCQ Regulate the Relationship Between CLIP and MHC-II on B Cells

The co-expression of CLIP and MHC-II on B cells was also examined following TLR and BCR activation. Both CpG and anti-IgM+IL-4 significantly increased the frequency of MHC-II^+^/CLIP^+^ B cells (*p* < 0.001, *p* < 0.001, respectively; Figure 2C). The induction of autophagy with RAPA significantly reduced the percentage of MHC-II^+^/CLIP^+^ B cells (*p* < 0.001) following CpG activation but not following anti-IgM+IL-4 activation (Figure 2F). Thus, RAPA reduced the expression of CLIP on the cell surface of CpG-activated B cells independent of its effects on MHC-II, while reductions in CLIP following BCR-mediated activation may be MHC-II-associated.

To further explore the effects of RAPA and HCQ on CLIP binding to MHC-II following TLR9 or BCR activation, the ratio of CLIP^+^ to CLIP- MHC-II^+^ B cells was assessed. In TLR9-activated cells, both RAPA and HCQ reduced the ratio of CLIP^+^ to CLIP- cells, resulting in an increase in cell-surface MHC-II without CLIP. In BCR-activated cells, RAPA and HCQ treatment did not alter this ratio (Figure 2M). These results further demonstrate the selectivity by which TLR9 and BCR activation of B cells leads to differences in B cell populations that express CLIP and MHC-II. Therefore, distinct mechanisms appear to regulate CLIP and MHC-II expression in response to TLR9 activation. It is also possible that MHC-II expression responds to CLIP levels, such that MHC-II is upregulated in response to reduced CLIP. This is supported by data showing that excess exogenous CLIP downregulates MHC-II [43,45]. Considering that RAPA reduces the percentage of MHC-II^+^ cells that express CLIP following TLR9 activation, it is possible that autophagy induction with RAPA promotes CLIP displacement from the antigen-binding groove of MHC-II.

### 2.4. RAPA and HCQ Regulate Loading of Antigenic Peptide on Activated B Cells

**Figure 3:**
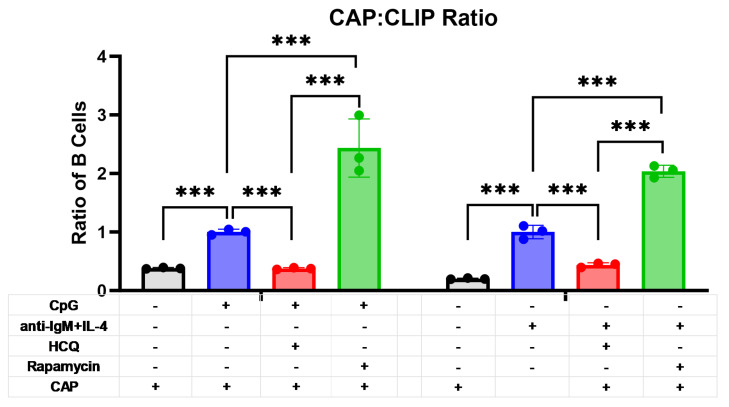
Displacement of CLIP and antigenic peptide loading into MHC-II. Splenocytes were cultured for 48 h, as described in Figure 1 and Figure 2. In response to TLR9 activation (**left**) and BCR activation (**right**), antigenic peptide (CAP) significantly increased in B cells. Co-stimulation with HCQ significantly decreased the loading of antigenic peptides, whereas RAPA significantly enhanced antigenic peptide loading into MHC-II. Data are presented as mean ± SEM, n = 3. *** *p* < 0.001.

Because RAPA reduced and HCQ increased cell-surface CLIP expression, it was hypothesized that loss of CLIP as a result of a RAPA would increase B cell antigenic peptide presentation. Following activation with CpG or anti-IgM+IL-4, along with either RAPA or HCQ, a CLIP antigenic peptide (CAP) [28] was added. RAPA significantly increased the CAP:CLIP ratio by approximately two-fold following stimulation with CpG (*p* < 0.001; Figure 3) or anti-IgM+IL-4 (*p* < 0.001; Figure 3). Conversely, HCQ significantly reduced the CAP:CLIP ratio in CpG-activated (*p* < 0.001; Figure 3) and anti-IgM+IL-4-activated (*p* < 0.001; Figure 3) cells. Thus, autophagy induction with RAPA appears to facilitate the displacement of CLIP, because there was an increase in the loading of antigenic peptide into the MHC-II groove. Conversely, the inhibition of autophagy with HCQ resulted in a reduction in the antigenic peptide loading into MHC-II.

## 3. Materials and Methods

### 3.1. Reagents

RPMI 1640 cell culture media (Fisher Scientific; Waltham, MA, USA, 11-835-030), heat-inactivated fetal bovine serum (FBS) (Fisher Scientific; Waltham, MA, USA, 10-082-147), 200 mM L-glutamine (Fisher Scientific; Waltham, MA, USA, 25-030-149), 1 M HEPES buffer (Fisher Scientific; Waltham, MA, USA, BP299100), ACK lysis buffer (VWR International; Radnor, PA, USA, 10128-802), gentamicin sulfate (Fisher Scientific; Waltham, MA, USA, 15-750-078), 100 mM sodium pyruvate (Fisher Scientific; Waltham, MA, USA, MT25000CI), phosphate-buffered saline (PBS) (Fisher Scientific; Waltham, MA, USA, 10-010-049), LIVE/DEAD^®^ fixable aqua dead cell stain (Fisher Scientific; Waltham, MA, USA, L34957), CellTrace™ Violet Cell Proliferation dye (Fisher Scientific; Waltham, MA, USA, C34557), penicillin–streptomycin (concentration of 10,000 units—10 mg/mL) (Sigma-Aldrich Inc.; St. Louis, MO, USA, P4333), 40 μm nylon cell strainers (Fisher Scientific; Waltham, MA, USA, NC2227717), TLR9 ligand CpG–oligodeoxynucleotide CpG–ODN 2006 (CpG) (InvivoGen Inc.; San Diego, CA, USA, tlrl-2006), F(ab’)2 fragment of anti-mouse IgM (Jackson ImmunoResearch Laboratories Inc.; West Grove, PA, USA, 115-006-020), and interleukin-4 (IL-4) (ThermoFisher Scientific; Waltham, MA, USA, 16-7048-81) were used.

### 3.2. Mice

C57BL/6 mice and B6-lpr mice with spontaneous mutation (Fas lpr) that are prone to autoimmune diseases were purchased from Jackson Laboratory Inc. (Bar Harbor, ME, USA) (Strain #000482). All animals were housed in the Baylor Scott and White Hospital vivarium facility under the IACUC guidelines, and all animal experiments were approved by Baylor Scott and White Hospital IACUC (Protocol #2013-021).

### 3.3. Splenocyte Isolation

Mice were anesthetized using isoflurane followed by cervical dislocation. Spleens were collected, washed in PBS containing 3% FBS (FBS/PBS), and passed through 40 μm nylon cell strainers to make single-cell suspensions. The red blood cells were lysed by incubating the cell pellet in ACK buffer for six minutes at room temperature (25 °C) in the dark. Cells were washed twice with FBS/PBS to remove traces of ACK buffer, and then the splenocytes were collected as a pellet and left on ice.

### 3.4. Peritoneal Cavity Lymphocyte Isolation

Mice were anesthetized using isoflurane and then euthanized in a CO_2_ chamber; then, 5 mL of ice-cold FBS/PBS was injected into the peritoneum of the animal, and after 90 s, the solution was collected using a Pasteur pipette. The collected FBS/PBS from the animal contained a mixture of B-1 and B-2 and other immune cells. The collected cells were washed once and were prepared for experimental setup.

### 3.5. Splenocyte Activation and Treatment

The isolated splenocytes were plated at a concentration of 1 × 10^6^ cells/mL in a Corning^®^ Costar^®^ 24-well flat bottom cell culture plates in RPMI 1640 cell culture media supplemented with heat-inactivated FBS at 5% final concentration (*v*/*v* %), 2 mM L-glutamine, 10 mM HEPES, 1mM sodium pyruvate, 100 units of penicillin, and 50 mg of streptomycin. Cells were cultured for 48 h, incubated in a water-jacketed CO_2_ incubator (ThermoFisher Scientific Inc., Waltham, MA, USA) at 5% CO_2_ concentration and 95% humidity. Splenocytes were activated with the TLR9 agonist CpG (5 µg/mL) or f(ab’)2 fragment of anti-mouse IgM + IL-4 (anti-IgM+IL-4) (5 ng/mL). We used a fragment of the antibody against the B cell antigen receptor instead of intact antibody as a surrogate for antigen receptor-mediated activation and to exclude Fc receptor engagement, which can result in a negative signal via the antigen receptor [33]. In subsequent experiments, splenocytes were cultured for 48 h with CpG or anti-IgM+IL-4 and concurrently treated with rapamycin (RAPA) (Selleckchem Inc.,; Houston, TX, USA, S1044; 10 µM), which activates autophagy, and hydroxychloroquine sulfate (HCQ) (Sigma-Aldrich Inc.; St. Louis, MO, USA, PHR1782-1G; 10 µM), which inhibits autophagy.

### 3.6. Cell-Surface Staining and Flow Cytometry

B cell subsets in the cultured splenocytes were evaluated by surface staining of the splenocytes with APC-Cy™7 anti-mouse CD19 (BD Biosciences Inc.; Franklin Lakes, NJ, USA, 553783), APC Anti-Mouse MHC Class II (BioLegend Inc.; San Diego, CA, USA, 107624), and FITC mouse anti-CLIP (15G4) (clone 15G4; Santa Cruz Biotechnology Inc.; Santa Cruz, CA, USA, sc-53946 FITC) along with LIVE/DEAD^®^ fixable aqua dead cell stain. For the staining of the B-1 B cells and B-2 B cells from the peritoneal cavity, the following antibodies were used: APC-Cy™7 rat anti-mouse CD19, and PE rat anti-mouse CD45R/B220 (BD Biosciences Inc.; Franklin Lakes, NJ, USA, 554881), PE-Cy™7 rat anti-mouse CD5 (BioLegend Inc.; San Diego, CA, USA, 100602), and Pacific Blue™ rat anti-mouse IgD (BioLegend Inc.; San Diego, CA, USA, 405711). All cells were analyzed on a Becton Dickson FACSCanto II flow cytometer (BD Biosciences Inc., San Jose, CA, USA), a 3-laser 10-parameter system with FACSDiva software (Version 8.0; BD Biosciences Inc., San Jose, CA, USA), and the flow data were analyzed using FlowJo^®^ software (Version 10.10; FlowJo, LLC, Ashland, OR, USA).

### 3.7. Proliferation Assay

The B cell proliferation assay was performed using CellTrace™ Violet Cell Proliferation dye. The basic principle of proliferation was based on dye dilution. For this experiment, we prepared a 5 mM stock solution of CellTrace™ Violet Cell Proliferation dye in dimethyl sulfoxide (DMSO). The isolated splenocytes from the mice were cleared of red blood cells by incubating with the ACK buffer followed by washing with FBS/PBS solution. For staining a pre-calculated number of cells, the splenocytes were resuspended in prewarmed PBS, and dye was added for a final concentration of 5 μM per 1 million cells per mL and incubated at 37 °C for 20 min in the dark; then, the cells were washed twice with FBS/PBS to quench unreacted dye. The stained cells were plated in a 24-well plate and stimulated with CpG or anti-IgM+IL-4 agonist with and without rapamycin.

### 3.8. Competitive Binding Studies

For competitive peptide binding studies, a target biotinylated peptide, CAP, with higher binding affinity for the MHC alleles than CLIP was synthesized using an algorithm that detects peptides with high binding affinity for MHC-II ([28], Elim Biopharma Inc.; Hayward, CA, USA). CAP was dissolved in DMSO at a concentration of 5 mg/mL. The splenocytes isolated from the C57BL/6 spleens were plated at a concentration of one million cells per well in 24-well plates in 1 mL of 5% FBS containing RPMI1640 growth media supplemented with standard amino acids. The cells were activated with CpG or anti-IgM+IL-4, as described above, and treated either with rapamycin or HCQ at a concentration of 10 μM along with the CAP peptide at a final concentration of 5 μg/mL. After 48 h of incubation in the CO_2_ incubator, the cells were washed and first stained with the surface markers for CD19, CD3, CLIP, and MHC-II antibodies and incubated on ice for 20 min, followed by two washes with FBS/PBS, and then stained with streptavidin to stain for the surface expression of CAP. Once the cells were analyzed, the data indicating the MFIs of CLIP and CAP were normalized, and the ratio of CAP to CLIP was calculated for each sample and plotted. A ratio above one indicates a greater amount of CAP than CLIP bound to MHC-II, suggesting that CAP displaced CLIP in the antigen-binding groove.

### 3.9. Statistical Analysis

The acquired data from the FlowJo^®^ software (Version 10.10) were saved in Microsoft Excel (Redmond, WA, USA) and further analyzed using GraphPad Prism (Version 10; La Jolla, CA, USA). Comparisons between the two groups (ex. CpG vs. CpG+Rapamycin) were performed using Student’s *t*-test. Comparisons of 3 or more groups were performed using one-way analysis of variance (ANOVA), and multiple comparisons were performed using the post hoc Holm–Sidak test. The statistical significance level was set at α = 0.05 for all tests, so *p* ≤ 0.05 was considered significant.

## 4. Conclusions

The current study demonstrates that modulating autophagy with RAPA or HCQ selectively regulates the expansion of B1 and B2 B cell subsets, CLIP^+^ B cells, CLIP and MHC-II on B cells, and antigenic peptide exchange with CLIP in the groove of MHC-II. As autophagy is induced by RAPA and inhibited by HCQ, these findings demonstrate that autophagy can be mediated to regulate distinct B cell subsets following TLR and BCR activation. This study also shows that autophagy modulates the displacement of CLIP and the subsequent loading of antigenic peptides into the MHC-II groove. As the in vivo physiological environment is more complex than in vitro, these conclusions should be confirmed in animal models to more fully understand the complex autophagic mechanisms involved. Nevertheless, these in vitro observations are supported by previous studies using the same B cell activation paradigms [28,46]. A second consideration is that the experiments rely on autophagy-modulating compounds HCQ and RAPA, which are likely to have additional effects on the signaling pathways being assessed. For example, HCQ inhibits TLR9 signaling, in part through its effects on the pH of the endosome in which DNA interacts with TLR9, independent of the effects on autophagy [47]. However, HCQ in the current study increased the expression of CLIP, despite the buffering effects of chloroquine in the lysosome. Future studies are needed to more comprehensively evaluate these putative autophagic mechanisms.

Although the current results did not include genetic models to manipulate autophagy genes, the data are consistent with a previous study showing that autophagy is required for B1 cell survival [15]. The data are also consistent with a previous study in which mice with a conditional Atg5 deletion had impaired antibody production in vivo and a reduced response to CpG in vitro [13]. Similarly, Acharya and colleagues [48] showed that loss of autophagy in B cells following Atg5 deletion had differential effects on the proliferation of marginal zone (MZ) B cells, depending on the method of B cell activation. The response to IgM was reduced, but the response to CpG DNA increased when autophagy was blocked [48]. Thus, the regulation of B1 and B2 B cell subsets may be an important mechanism for restricting or promoting MHC-II antigen presentation by B cells, regulating the overall B cell inflammatory environment. These findings have important implications for a more selective approach to depleting specific B cell subsets.

## Data Availability

Data for this study is available from the corresponding authors upon reasonable request.

## Abbreviations

The following abbreviations are used in this manuscript:

BCR	B cell receptor
CAP	CLIP antagonist peptide
CD74	Invariant chain
CLIP	Class II-associated invariant peptide
CpG	Cytidine monophosphate guanosine oligodeoxynucleotides
FBS	Fetal bovine serum
HCQ	Hydroxychloroquine
MHC-II	Major histocompatibility complex II
MFI	Mean fluorescent intensity
mTOR	Mammalian target of rapamycin
PBS	Phosphate-buffered saline
TLR	Toll-like receptor
